# On the Maintenance of Expectations in Major Depression – Investigating a Neglected Phenomenon

**DOI:** 10.3389/fpsyg.2017.00009

**Published:** 2017-01-18

**Authors:** Tobias Kube, Winfried Rief, Julia A. Glombiewski

**Affiliations:** Department of Clinical Psychology and Psychotherapy, Philipps-University of MarburgMarburg, Germany

**Keywords:** major depression, expectation violation, expectancy, immunization, self-concept, expectation persistence, cognitive-behavioral therapy, behavioral experiment

## Abstract

In this perspective paper, we suggest that among patients suffering from major depressive disorder (MDD), dysfunctional expectations are maintained despite experiences that are contrary to these expectations. Surprisingly, this persistence of expectations in MDD has not yet been addressed by empirical studies. We argue that it is worthwhile to investigate this phenomenon with the aim of improving the treatment of MDD, and we provide a theoretical framework for understanding it. It is hypothesized that the persistence of expectations is primarily due to a process called immunization. That is, people experiencing depressive symptoms may cognitively reappraise the contradictory experience such that expectations do not need to be changed. There may be two mechanisms underlying this immunization: (1) the experience in the expectation-violating situation is considered to be an exception; or (2) the credibility of the information gained from the experience is called into question. Moreover, the maintenance of expectations may be particularly persistent if a person’s expectations reflect his or her self-concept, as self-concept has been shown to be associated with future expectations. To empirically examine the hypothesized maintenance of expectations in MDD, we propose an experimental approach which could provide important implications for the treatment of MDD within cognitive behavioral therapy. We suggest that psychological interventions such as behavioral experiments should more rigorously focus on patients’ appraisal of expectation-violating experiences in order to prevent immunization processes. Therapists should continuously examine whether patients’ expectations were modified and should address the reasons for the maintenance of expectations.

## The Relevance of Expectations in Major Depression

In a clinical psychology framework, expectations^1^ have been defined as future-directed cognitions that focus on the incidence or non-incidence of a specific event or experience (Kube et al., 2016). Based on the Rescorla–Wagner model (Rescorla, 1967), expectations are developed through learning processes (Cleeremans and McClelland, 1991; Colloca and Benedetti, 2009; Colloca and Miller, 2011). Expectations have been identified to contribute substantially to clinical outcome in various medical conditions (Auer et al., 2016; Nestoriuc et al., 2016). Moreover, expectations have been shown to be one of the major components contributing to placebo and nocebo responses in clinical trials (Rief et al., 2008, 2011; Schwarz et al., 2016), and expectations can substantially enhance the effects of drug-specific components (see Kube and Rief, 2016 for a review). With regard to antidepressant clinical trials, large placebo effects have been reported (Kirsch and Sapirstein, 1998; Kirsch et al., 2002, 2008, Rief et al., 2009), and they are assumed to be mainly based on expectation mechanisms (Shedden Mora et al., 2011; Rutherford et al., 2016). Given the great impact of expectancies in clinical research, Rief et al. (2015) have discussed expectancies as core features of mental disorders (Rief et al., 2015). For major depressive disorder (MDD), there is evidence that people suffering from MDD hold situation-specific dysfunctional expectations which may be elicited by depressive core beliefs (Kube et al., 2016). Clinical observations suggest that these expectations are maintained despite experiences that are contrary to patients’ expectations (“expectation violation”) (Rief and Glombiewski, 2016). Surprisingly, this observed persistence of expectations in MDD has not yet been investigated in empirical studies. In this perspective article, we argue that it is worthwhile to investigate the maintenance of expectations in MDD, and we provide a theoretical framework for it with the aim of inspiring empirical research into this neglected phenomenon. This could help to develop psychological interventions aiming at enhancing expectation change and could thus substantially improve current cognitive behavioral treatment (CBT) of MDD.

Exposure therapy for the treatment of anxiety disorders has recently focused on disconfirming disorder-specific expectations by maximizing the discrepancy between patients’ expectations and actual situational outcomes in expectation-violating situations, which is discussed as promising approach to modify patients’ expectations and thereby reduce anxiety symptoms (Craske et al., 2014; Craske, 2015). In MDD, however, disorder-specific expectations are less obvious: people suffering from MDD often report somatic symptoms (such as sleep disturbance, loss of appetite etc.) and negative mood, but may be less aware of cognitions such as expectations (Beck, 2011). Prior research has indicated that (treatment) outcome expectations (Greenberg et al., 2006; Price et al., 2008), self-efficacy expectancies (Ludman et al., 2003; Gopinath et al., 2007; Gordon et al., 2011), and global expectations about future events (Strunk et al., 2006; Vilhauer et al., 2012) predict the course of depressive symptoms. However, situation-specific expectations resulting from depressive core beliefs have received limited attention in psychotherapy research. Similarly, CBT of MDD has primarily focused on present-focused cognitions and automatic thoughts by using cognitive and behavioral interventions (such as cognitive restructuring and behavioral experiments), while rigorously disconfirming future-directed expectations has so far received less attention. A more focused examination of patients’ expectations may be advantageous for optimizing psychological interventions (Rief and Glombiewski, 2016).

This is especially important because MDD has been shown to have a high relapse rate (Judd et al., 1998; Lin et al., 1998; Solomon et al., 2000; Pintor et al., 2003; Eaton et al., 2008; Moffitt et al., 2010). According to Risch et al. (2012), relapse may be due to the reactivation of dysfunctional thoughts when confronted with new stressful events. Moreover, a substantial group of patients does not respond to usual CBT (Hofmann et al., 2012; Button et al., 2015; Beard et al., 2016). We hypothesize that the long-term efficacy of CBT could be increased by more rigorously addressing the mechanisms underlying the persistence of dysfunctional expectations. Before discussing these clinical implications, we first address in more detail the phenomenon of expectation persistence.

## Frameworks for the Maintenance of Expectations in Expectation-Violating Situations

Rief et al. (2015) proposed a theoretical model to explain the development and maintenance of expectations. According to this model, expectations are shaped by learning processes, as well as by social influences and individual differences. After being confronted with experiences that are contrary to one’s expectations, expectations can either be changed or maintained (Rief et al., 2015). We suggest that healthy individuals are able to change their expectations after expectation-violating experiences. For instance, though many people may initially expect to fail when attempting a novel difficult task, healthy individuals may modify their expectations about future performance after receiving feedback indicating that they performed well. However, we suggest that among individuals suffering from MDD expectations are often maintained despite experiences that are contrary to their expectations. We argue that this persistence of expectations despite contradictory experiences is a core feature of MDD, and that the maintenance of expectations in MDD is due to maladaptive information processing involving a process called “immunization.”

### Immunization as Important Mechanism for the Persistence of Expectations

The term “immunization” was originally introduced by Brandstädter and Greve (1994) in a developmental psychology framework and needs to be distinguished from its use in a medical context. According to Brandstädter and Greve (1994), immunization serves as self-protective mechanism by reappraising experiences of loss in a self-worth stabilizing manner. In clinical psychology, however, immunization has not yet been empirically investigated, and little is known about this phenomenon. According to Rief et al. (2015), in a clinical psychology framework, immunization means that an expectation-violating experience is cognitively reappraised so that one’s prior expectation is confirmed by a *post hoc* evaluation, while the contradictory experience is discounted. We suggest that there are two possible mechanisms underlying this immunization process. First, the experience gained in the expectation-violating situation may be considered to be an exception rather than the rule. For instance, a person might maintain expectations of failure after successful experiences by thinking, “Well, I managed that, but it was an easy task.” and thus reappraising the contradictory experience. Second, a person may question the credibility of the information gained in an expectation-violating situation. For instance, the expectation “Nobody will be there for me when I ask for help” may be maintained despite another person’s offer of help by a reappraisal such as, “He only helped me because he wanted to get rid of me afterward. In fact, he does not like me and is not interested in how I am feeling.” Both mechanisms may lead to a persistence or possibly even reinforcement of expectations via cognitive reappraisal of the contradictory experience in a way that confirms prior expectations. In addition to this immunization process, other forms of maladaptive information processing in MDD, such as cognitive distortion, selective attention or selective memory (Beck, 1963; Hammen and Krantz, 1976; Hammen, 1978; Beck et al., 1979; Krantz and Hammen, 1979; Haaga and Beck, 1995; Beck and Haigh, 2014), may contribute to the maintenance of expectations.

### A Social Psychology Perspective

The idea that individuals reappraise contrary information to experience cognitive consistency is supported by research from social and cognitive psychology (Lord et al., 1979; Ross and Lepper, 1980; Frey and Rosch, 1984; Oaksford and Chater, 2007). Cognitive consistency theories and especially the theory of cognitive dissonance (Festinger, 1962) have impacted research on how individuals change cognitions and attitudes. According to Festinger (1962), cognitive dissonance is an aversive state that is generated when a person has two or more contrary cognitions. As a result, people aim to reduce this dissonance by changing one or more of the inconsistent cognitions.

**FIGURE 1:**
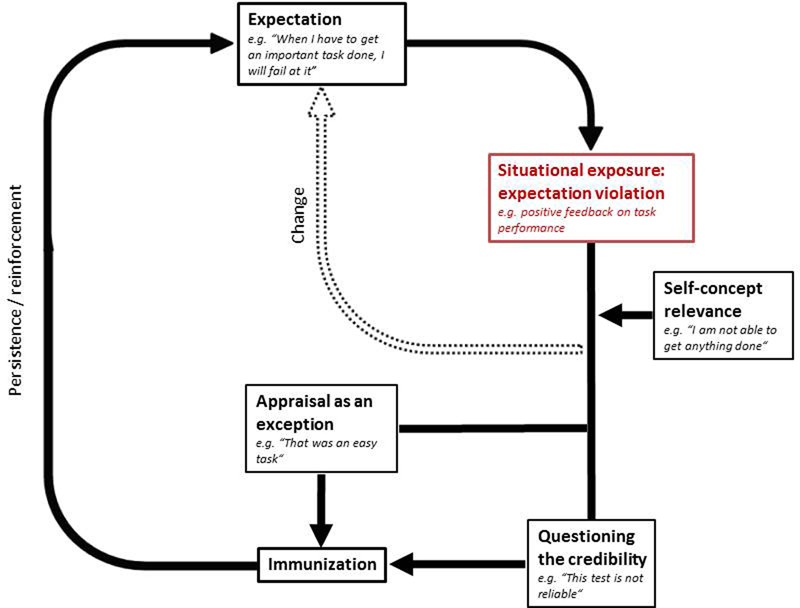
**The hypothesized model of expectation persistence in MDD with immunization as an underlying mechanism**.

Moreover, research from social and personality psychology has provided extensive evidence that a person’s self-concept remains quite stable over time, as individuals selectively search for information that confirms the self-concept while denying self-concept incongruent information (Markus, 1977; Swann and Read, 1981a,b; Swann and Hill, 1982; Markus and Wurf, 1987). Hence, people seem to be prone to a “confirmation bias,” and they are supposed to use “positive test strategies,” meaning that one prefers to use strategies that are considered to confirm the prior hypothesis (Klayman and Ha, 1987). More specifically, McFarlin and Blascovich (1981) demonstrated in an experimental study that an individual’s level of self-esteem predicts expectations about future performance, irrespective of feedback on performance. Given that MDD is associated with low self-esteem (Lewinsohn et al., 1988; Roberts and Monroe, 1992, 1994; Joiner et al., 1999; Orth et al., 2008), we suggest that self-esteem or other aspects of an individual’s self-concept may be moderator variables within the immunization process. That is, the maintenance of expectations via immunization is more likely if the expectations involved are closely related to one’s self-concept. For instance, the expectation “When I have to get an important task done, I will fail at it” may be particularly persistent if an individual’s self-concept includes the assumption “I am not able to adequately cope with performance-related situations.” This may be the case in individuals suffering from MDD, since people experiencing depressive symptoms are thought to hold dysfunctional core beliefs such as, “I am not able to get anything done” (Beck et al., 1979; Beck, 2011). **Figure 1** illustrates the suggested immunization process while taking into account the self-concept relevance of expectations.

Also, we suggest that the maintenance of self-concept related expectations is facilitated by the fact that actively modifying one’s expectations is perceived as more effortful than reappraising the experience, since one thereby does not need to change one’s self-concept (see also Swann and Hill, 1982). For instance, if an individual were to change the expectation, “When I have to get an important task done, I will fail at it” into “When I have to get an important task done, I will manage it,” it would follow that the individual is abandoning an excuse for not exposing oneself to performance-related situations. Our clinical experiences, however, suggest that people experiencing depressive symptoms tend to use their pessimistic expectations as justification for withdrawal and avoidance (e.g., “I do not need to try that because I will fail at it anyway”). For instance, modifying one’s expectation to “I will be able to manage that” may imply that one has the responsibility to overcome existing challenges and is no longer able to use expectations about failure as excuse for withdrawal and avoidance. This may threaten the self-concept against the background of past behavior, hence facilitating expectation maintenance rather than expectation change.

### A Neurobiological Perspective

Expectations have been suggested to shape experiences and to affect how an individual experiences its environment (Kirsch, 1999). This idea has recently been examined by cognitive neuroscience researchers. For instance, it has been shown that prior expectations bias stimulus processing in the visual cortex (Kok et al., 2013). Additionally, research from cognitive neuroscience has indicated that expectation-violating effects (e.g., by using invalid cues) can lead to a “surprise-attention link,” resulting in a shift of attention, which may hinder or facilitate learning processes (Horstmann, 2015). Given the maladaptive information processing in MDD, this bias in experiencing one’s environment by prior expectations could be especially pronounced in people suffering from MDD, which could further contribute to expectation maintenance.

## Investigating the Persistence of Expectations

To empirically examine the hypothesized phenomenon of expectation maintenance in MDD, we propose a stepwise experimental approach (see **Table 1**). First, researchers should attempt to empirically examine the clinical observation that people suffering from MDD tend to maintain their expectations despite expectation-violating experiences. For this purpose, researchers could focus on explicit expectation regarding personal achievement (e.g., “I will be successful in working on an unknown test”), and they could ask participants to complete an unknown test which is said to be very difficult. Then, participants could be given standardized performance feedback that is surprisingly positive. Thereby, it could be examined whether subjects changed their initial expectations after receiving expectation-violating feedback; that is, the possible change of expectations from pre to post would be the dependent variable. At the same time, the hypothesized immunization process as an underlying mechanism could be examined by exploring the reasons for expectation change vs. expectation maintenance.

**Table 1:** Proposed stepwise procedure for the investigation of expectation persistence.

	Aim of the investigation step
Step 1	Systematically observing that people suffering from MDD relative to healthy controls tend to more frequently maintain their expectations despite experiences contrary to expectations. Developing an experimental paradigm for the investigation of expectation violation in MDD. Developing a questionnaire assessing situation-specific expectations in MDD.
Step 2	Experimentally manipulating the appraisal of an expectation-violating situation and thus experimentally manipulating immunization.
Step 3	Examining the self-concept relevance of expectations as a possible moderator of immunization in correlational analyses. Subsequently, experimentally manipulating the self-concept relevance of expectations.
Step 4	Conducting a clinical study with cognitive behavior therapy enhanced with expectation focused psychological interventions vs. treatment as usual.

After this exploratory approach, it may be useful to experimentally manipulate the appraisal of the expectation-violating situation to impede or enhance immunization. For this purpose, experimenters could vary whether or not participants are guided to consider the expectation-violating experience as exceptional. For instance, one could provide standardized information to participants suggesting that the test completed either is or is not useful for predicting achievement in other situations. Thus, it can be examined to what degree the manipulation of the perceived relevance of the expectation-violating experience influences expectation change. Another approach for experimentally manipulating immunization could be the induction of self-focused rumination vs. distraction after an expectation-violating situation. Based on Lyubomirsky et al.’s (2003) paradigm, it is hypothesized that self-focused rumination in individuals with MDD triggers negative thoughts about perceived past failures, which may facilitate immunization and may therefore additionally contribute to expectation maintenance. To investigate self-concept relevance as a possible moderating variable, correlational analyses could examine whether expectation maintenance is more likely if the expectations are closely related to the individual’s self-concept. If correlational analyses yield promising results, researchers could experimentally vary whether or not the expectations examined in the study are associated with self-concept. Finally, clinical studies might examine whether enhancing CBT with expectation focused interventions (see also Rief and Glombiewski, 2016) increases therapy success relative to treatment as usual.

## Clinical Implications

A better understanding of the persistence of expectations in MDD would have several implications for CBT for MDD. Within CBT for MDD, behavioral experiments are an effective method of testing automatic thoughts in order to facilitate cognitive restructuring (Dobson and Hamilton, 2003; Beck, 2011; Dobson, 2016). Given the relevance of disorder-specific expectations in MDD, we encourage therapists to more specifically focus on patients’ expectations when designing behavioral experiments, as the “if-then” structure of expectations (as opposed to other automatic thoughts) makes them susceptible to falsification (Kube et al., 2016). That is, behavioral experiments can serve as expectation-violating situations insofar as patients can gain experiences that are contrary to their expectations (Craske et al., 2014). However, clinical experiences suggest that experiences contrary to patients’ expectations do not always result in successful change of expectations (Rief and Glombiewski, 2016). In such cases, it may be worthwhile to actively explore the reasons for the maintenance of expectations in order to impede immunization processes, which could improve therapy success in multiple ways.

First, if a patient considers the experience in a behavioral experiment to be an exception, the therapist should discuss whether this appraisal is accurate or useful. If necessary, behavioral experiments may subsequently be repeated under different circumstances to call the patient’s appraisal into question. Thus, the generalizability of the experience gained in a behavioral experiment should be emphasized to prevent immunization processes. Second, if a patient fundamentally questions the credibility of the experience, the therapist might help the patient to re-examine the validity of the experience. Third, therapists should carefully consider whether the expectations tested in a behavioral experiment are closely related to the patient’s self-concept, and should be aware that if so, change in expectations may be less likely. Such awareness may prevent disappointment for both patient and therapist, and the therapist can motivate the patient to change his or her behavior, e.g., by discussing the consequences of the behavior. Fourth, in addition to exploring the reasons for maintenance of expectations *after* a behavioral experiment, it may be useful to discuss with the patient the conditions under which he/she would change his/her expectations *before* engaging in the behavioral experiment. This would allow the therapist and patient to agree on the conditions for the behavioral experiment such that the patient would consider a violation of his/her expectations to be a valid experience. This procedure might help to prevent *post hoc* confirmation of expectations via immunization.

Given the high relapse rates in MDD (Judd et al., 1998; Lin et al., 1998; Solomon et al., 2000; Pintor et al., 2003; Eaton et al., 2008; Moffitt et al., 2010), rigorously addressing patients’ expectations may be helpful with respect to long-term benefit from therapy, as patients can be encouraged to test future dysfunctional expectations independently after therapy completion. If CBT were to enable patients to prevent dysfunctional immunization processes, this could result in additional positive experiences which in turn could impede the reactivation of dysfunctional thoughts (Risch et al., 2012).

Considering the maintenance of expectations may also be useful for the treatment of other mental disorders. Modifying patients’ expectations through exposure to expectation-violating situations has been discussed as a promising approach in the treatment of anxiety disorders (Craske et al., 2014; Craske, 2015), obsessive compulsive disorders (Craske et al., 2014), and chronic pain (Riecke et al., 2013). We believe that impeding immunization processes (as discussed for MDD in this article) might also be an important mechanism of change in these disorders. Thus, we hope that the proposed theoretical model for the persistence of expectations will inspire future research with the aim of optimizing cognitive-behavioral therapy by preventing immunization processes not only in MDD, but also in other mental disorders involving dysfunctional expectations.

## Conclusion

The maintenance of expectations despite experiences that are contrary to expectations is believed to be a core feature of MDD. We suggest that this persistence of expectations is due to maladaptive information processing in MDD, in particular, immunization processes. Immunization is hypothesized to be especially pronounced if an individual’s expectations are closely associated with his or her self-concept. This should be examined in a series of experimental studies and could provide useful information for the treatment of depression. Carefully addressing the reasons for expectation persistence may be useful for optimizing psychological interventions, hence increasing the long-term efficacy of CBT.

## Author Contributions

TK: Did the major part of the work with regard to conception and design; mainly contributed to the development of the manuscript; approves the manuscript to be published; agrees on being accountable for all aspects of the work in ensuring that questions related to the accuracy or integrity of any part of the work are appropriately investigated and resolved. WR: Substantially contributed to the conception of the work; revised the manuscript critically for important intellectual content; approves the manuscript to be published; agrees on being accountable for all aspects of the work in ensuring that questions related to the accuracy or integrity of any part of the work are appropriately investigated and resolved. JG: Substantially contributed to the conception of the work; revised the manuscript critically for important intellectual content; approves the manuscript to be published; agrees on being accountable for all aspects of the work in ensuring that.

## Conflict of Interest Statement

The authors declare that the research was conducted in the absence of any commercial or financial relationships that could be construed as a potential conflict of interest.

## Footnotes

1 The terms ‘expectation’ and ‘expectancy’ are often used in an interchangeable way. However, ‘expectation’ is more frequently used as a specific, verbalized construct whereas ‘expectancies’ may be present without full awareness (i.e., implicit expectancies). In this manuscript, we only use the term ‘expectation.’
